# Aquaporin-6 May Increase the Resistance to Oxidative Stress of Malignant Pleural Mesothelioma Cells

**DOI:** 10.3390/cells11121892

**Published:** 2022-06-10

**Authors:** Giorgia Pellavio, Simona Martinotti, Mauro Patrone, Elia Ranzato, Umberto Laforenza

**Affiliations:** 1 Human Physiology Unit, Department of Molecular Medicine, University of Pavia, 27100 Pavia, Italy; giorgia.pellavio@unipv.it; 2 DiSIT-Dipartimento di Scienze e Innovazione Tecnologica, University of Piemonte Orientale, viale Teresa Michel 11, 15121 Alessandria, Italy; simona.martinotti@uniupo.it (S.M.); mauro.patrone@uniupo.it (M.P.); elia.ranzato@uniupo.it (E.R.)

**Keywords:** hydrogen peroxide, peroxiporins, epithelioid, biphasic, tumor proliferation, gene silencing, Hyper7 probe

## Abstract

Malignant pleural mesothelioma (MPM) is an aggressive cancer of the pleural surface and is associated with previous asbestos exposure. The chemotherapy drug is one of the main treatments, but the median survival ranges from 8 to 14 months from diagnosis. The redox homeostasis of tumor cells should be carefully considered since elevated levels of ROS favor cancer cell progression (proliferation and migration), while a further elevation leads to ferroptosis. This study aims to analyze the functioning/role of aquaporins (AQPs) as a hydrogen peroxide (H_2_O_2_) channel in epithelial and biphasic MPM cell lines, as well as their possible involvement in chemotherapy drug resistance. Results show that AQP-3, -5, -6, -9, and -11 were expressed at mRNA and protein levels. AQP-6 was localized in the plasma membrane and intracellular structures. Compared to normal mesothelial cells, the water permeability of mesothelioma cells is not reduced by exogenous oxidative stress, but it is considerably increased by heat stress, making these cells resistant to ferroptosis. Functional experiments performed in mesothelioma cells silenced for aquaporin-6 revealed that it is responsible, at least in part, for the increase in H_2_O_2_ efflux caused by heat stress. Moreover, mesothelioma cells knocked down for AQP-6 showed a reduced proliferation compared to mock cells. Current findings suggest the major role of AQP-6 in providing mesothelioma cells with the ability to resist oxidative stress that underlies their resistance to chemotherapy drugs.

## 1. Introduction

Malignant pleural mesothelioma (MPM) is a type of cancer of the mesothelium from the serosal surface of the body. It is a tumor as rare as it is aggressive and, in Italy, it represents 0.4% of tumors in males and 0.2% in females. The main cause of this tumor is exposure to asbestos (occupational or, more rarely, environmental or domestic exposure) and rarely other causes, such as exposure to erionite (a zeolites mineral), chest and abdomen radiation, inhalation of other fibrous silicates, and intrapleural thorium dioxide as a contrast medium. The main symptoms of MPM are shortness of breath and chest wall pain due to the fluid accumulation in the chest or the abdomen [1]. The incidence of mesothelioma seems to have reached a plateau in some countries, such as the USA (approximately 3200 per year). However, the incidence in many European countries is not expected to peak before the 2020s because of the long latency period between asbestos exposure and diagnosis (up to 30–50 years), and the extensive use of asbestos up until the 1970s in most high-income countries. Moreover, asbestos is still not banned worldwide, and its extraction and use are still ongoing in many countries (e.g. China, India, Kazakhstan, Russia). According to 2013 WHO predictions, the continued use of asbestos could be responsible for an epidemic of asbestos-related diseases in the upcoming decades [2]. Histologically, three subtypes of malignant mesothelioma can be considered: 1) epithelioid: the most common (60–70%) which has better prognoses; 2) biphasic: characterized by both epithelioid and sarcomatous areas, represents about 30% of mesotheliomas; and 3) sarcomatous: makes up approximately 10% of mesotheliomas. It has not been studied extensively and represents the one with the poorest prognosis.

The MPM has a very poor prognosis due to the low response to common medical treatments, and the median survival is approximately 6 to 12 months [3]. The median survival for patients in the best prognostic group of about 30 months compared with 1.8 months for patients in the worst prognostic group [4].

Unfortunately, no reliable prognostic markers were clinically used. Until now, the most important prognostic factors were the stage and size of cancer (whether the tumor can be removed completely by surgery), the amount of fluid in the chest or abdomen, age, sex, the patient’s activity level and general health, the histological subtype, WBC count, and hemoglobin concentration [1].

Aquaporins (AQPs) are integral membrane proteins that function as bidirectional water-selective channels but have been found to play a role in important cellular functions, such as cell proliferation, cell differentiation, cell migration, and cell adhesion. Thirteen water channel proteins have been identified in mammals and, based on their permeability, structure, and characteristics, they have been divided into three groups: (i) aquaporins (AQP0, 1, 2, 4, 5, 6, and 8) selectively permeable to water; (ii) aquaglyceroporins (AQP3, 7, 9, and 10) permeable to glycerol, urea, and other small solutes in addition to water; (iii) S-aquaporins (AQP11 and 12), with peculiar intracellular localization and functions, not yet fully clarified [5,6,7]. A fourth group, peroxiporins, includes paralogs belonging to the three groups mentioned above: AQP0, 1, 3, 5, 8, 9, and 11. These AQPs have high hydrogen peroxide (H_2_O_2_) permeability and play an important role in ROS scavenging [8,9,10,11,12,13,14,15,16,17].

In the last decade, compelling evidence for the key role of AQPs in tumor biology, including tumor-associated edema, tumor cell migration, tumor proliferation, and tumor angiogenesis, has emerged [18,19]. For these reasons, AQPs have been indicated as a possible target for cancer treatment [20,21].

In rodents and humans, AQP1 is expressed in the mesothelial cells of the pleura and peritoneum [22,23] with localization at the apical pole [24]. AQP1-null mice studies revealed the main role of pleural water transport across mesothelial cells in keeping with its apical membrane localization [25]. In addition to mesothelial cells, AQP1 was also found in vascular endothelium [26].

The presence of pleural effusions in MPM has led to the hypothesis of an involvement of AQP1 in the disease. Klebe and coworkers have demonstrated that AQP1 has a functional role in MPM proliferation, movement, and anchorage-independent growth [27]. The specific blockade of AQP1 in mesothelioma using a pharmacological blocker (AqB050) or using a specific AQP1-siRNA decreased cell proliferation, motility, and metastatic potential in vitro but not in vivo [27]. In vitro, MPM AQP1 inhibition can reduce cell adhesion, migration, and tumor sphere formation in an extracellular component type and histological type-dependent manner [28].

AQP1 has also been proposed as a possible prognostic factor with higher AQP1 expression levels that have been related to increased survival in MPM patients [24,29]. Concerning mesothelioma subtypes, the authors showed that the AQP1 staining pattern decreases as the survival period of these patients decreases: sarcomatoid < biphasic < epithelioid [24]. It has been proposed that the reduced AQP1 expression is an indication of tumor de-differentiation [29].

The treatment of MPM is complex and the survival outcomes rather than the overall survival data are, to date, disappointedly daunting. AQPs were widely studied in the hope of discovering possible prognostic markers and new potential therapeutic targets.

This study aims to investigate the peroxiporins role in MPM cell lines of epithelioid and biphasic histotypes. For this purpose, we determined: (1) the expression of AQP mRNAs and proteins; (2) the cellular localization; (3) the water permeability features in oxidative stress conditions; and (4) the contribution of AQP6 in water and H_2_O_2_ permeability and proliferation by selective gene silencing of MPM cells.

## 2. Materials and Methods

### 2.1. Cell Culture

Experiments were carried out on both mesothelioma and normal mesothelial immortalized cell lines. The following human cell lines were used: MeT-5A (a human non-malignant mesothelial cell line) [30], as well as REN [31] and MSTO-211H [32] (epithelioid and biphasic MPM cells, respectively). Cells were routinely grown in plastic tissue culture flasks using Dulbecco’s modified minimal essential medium–high glucose, supplemented with 10% fetal bovine serum, 1% L-glutamine, 1% penicillin, and streptomycin, and maintained at 37 °C in a humidified atmosphere of 5% CO_2_, 95% air.

### 2.2. RNA Isolation and RT-qPCR

Total RNA was extracted from MeT-5A, REN, and MSTO-211H cells using the QIAzol Lysis Reagent (QIAGEN, Milan, Italy). Reverse transcription was performed using MMLV reverse transcriptase M1701 (Promega, Milano, Italy), as previously described [33]. The primers used for amplification are specific for AQP2, 3, 4, 5, 6, 7, 8, 9, 10, and 11, and are listed in Table 1. Briefly, QuantiFast SYBRGreen PCR Master Mix (Qiagen, Milan, Italy) was used to perform the qPCR. The qPCR protocol consisted of an initial denaturation of 5 min at 95 °C followed by 40 cycles of denaturation at 95 °C for 10 s, annealing (see Table 1), and extension at 60 °C for 30 s. Reverse transcription was always performed, either in the presence (positive) or in the absence (negative control) of the reverse transcriptase enzyme. The qPCR reactions were normalized using β-actin or beta-2-microglobulin (B2M) as housekeeping genes (Table 1). Melting curves were generated to detect the melting temperatures of specific products immediately after the PCR run. The triplicate threshold cycles (Ct) values for each sample were averaged resulting in mean Ct values for both the gene of interest and the housekeeping gene. The gene Ct values were then normalized to the housekeeping gene by taking the difference: ΔCt = Ct(AQP gene) – Ct(housekeeping gene). ΔCt values of REN and MSTO-211H were subtracted of ΔCt values of Met-5A to obtain the ΔΔCt values. In AQP6 silencing experiments, ΔCt values of siRNA cells were subtracted of ΔCt values of Ctr cells. The fold change values were expressed as 2^−ΔΔCt^. PCR products were separated on a 3% Nusieve® (2:1) gel agarose, stained with ethidium bromide, and acquired with the Image Master VDS (GE Healthcare, Milano, Italy). The molecular weight of the PCR products was compared with the DNA molecular weight marker VIII (Roche Molecular Biochemicals, Monza, Italy).

### 2.3. Immunoblotting

Cells were homogenized in RIPA buffer (150 mM NaCl, 0.5% sodium deoxycholate, 0.1% SDS, 0.1% Triton X-100, 50 mM Tris-HCl, pH 8), supplemented with a protease inhibitor cocktail (cOmplete Tablets EASYpack, 04693116001; Merck KGaA, Darmstadt, Germany). Homogenates were solubilized in Laemmli buffer, and 30 µg proteins was separated on precast gel electrophoresis (4–20% Mini-PROTEAN TGX Stain-Free Gels, Bio-Rad, USA) and transferred to the PVDF membrane (Trans-Blot Turbo Transfer Pack, #1704156, Bio-Rad, USA) with the Trans-Blot Turbo Transfer System (#1704150, Bio-Rad, Segrate (MI), Italy). The membranes were blocked for 1 h in Tris-buffered saline containing 5% non-fat dry milk and 0.1% Tween (blocking solution). Membranes were incubated overnight with anti-AQP3 rabbit polyclonal IgG (ab125045, 1:1000; Abcam, Cambridge, UK), anti-AQP5 rabbit polyclonal IgG (A4985, 1:1000; Merck, Milan, Italy), and anti-AQP6 rabbit polyclonal IgG (# AQP61-A, 1:1000; Alpha Diagnostic International, San Antonio, TX, USA) in blocking solution. The membranes were washed and incubated for 1 h with goat anti-rabbit IgG antibody, which was peroxidase-conjugated (AP132P; Millipore part of Merck S.p.a., Vimodrone, Italy) and diluted 1:100,000 in the blocking solution. The bands were detected with the Westar Supernova Western blotting detection system (CYANAGEN, Bologna, Italy). Pre-stained molecular weight markers (ab116028, Abcam, Cambridge, UK) were utilized to calculate the band molecular weights. Blots were stripped [34] and reprobed with anti-β-actin rabbit polyclonal IgG (AB-81599, 1:2000; Immunological Sciences, Rome, Italy) or anti-β-2-microglobulin rabbit monoclonal (ab75853, 1:10000; Abcam, Cambridge, UK) diluted in blocking solution. Densitometry was performed by acquiring the blots with the iBrightTM CL1000 Imaging System (Thermo Fisher Scientific, Monza (MB), Italy). The semi-quantitation analysis of the bands was performed using the iBA (iBright Analysis Software; Thermo Fisher Scientific, Monza (MB), Italy) and the results were expressed as AQP/ β-actin or AQP/ β-2-microglobulin ratio.

### 2.4. Immunolocalization

AQP localization in MPM cells was studied using immunocytochemistry and double immunofluorescence techniques. Confluent MPM cells grown on coverslips were fixed with freshly prepared 4% paraformaldehyde in PBS for 30 min in a Petri dish and washed in PBS unless otherwise stated.

#### 2.4.1. Immunocytochemistry

Cells were treated with 0.3% hydrogen peroxide for 10 min at room temperature to block the endogenous peroxidases. After washing for 10 min with PBS, cells were blocked with 3% BSA in PBS for 30 min at room temperature. Coverslips were incubated overnight at 4 °C with affinity pure primary antibodies: anti-AQP3 rabbit polyclonal IgG (ab125045, 1:400; Abcam, Cambridge, UK), anti-AQP5 rabbit polyclonal IgG (A4985, 1:500; Merck, Milan, Italy), and anti-AQP6 rabbit polyclonal IgG (# AQP61-A, 1:1000; Alpha Diagnostic International, San Antonio, TX, USA), diluted in PBS. After three 10 min washes with PBS, coverslips were incubated for 30 min at room temperature with goat anti-rabbit IgG HRP-conjugated (ab236466, Mouse and Rabbit Specific HRP/DAB IHC Detection Kit, Micro-polymer, Abcam, Cambridge, UK). The reaction was visualized by incubation with a DakoCytomation 3,3’-diaminobenzidine chromogen solution. The cells were counterstained with hematoxylin, dehydrated, and mounted in Leica CV Mount (14046430011, Leica biosystems, Buccinasco (MI), Italy). Control experiments were performed by omitting the primary antibody. Then, coverslips were examined by light microscopy using an Olympus BX41, and the digital images were acquired with the Nikon DS-Fi1 digital camera using Nis Element F Imaging Software (2.33, Nikon, Tokyo, Japan).

#### 2.4.2. Double Immunofluorescence

Immunolocalization of AQP6 was evaluated in MeT-5A, REN, and MSTO-211H cells, which were cultured on coverslips, and incubated with 50 μg/mL concanavalin A FITC labeled (C7642, Merck, Milan, Italy) in PBS for 1 h at room temperature. After three washes with PBS, the cells were fixed in 4% paraformaldehyde in PBS for 30 min and then washed with PBS. The cells were then blocked with 3% BSA in PBS at room temperature for 30 min. Coverslips were incubated overnight at 4 °C with affinity pure anti-AQP6 rabbit polyclonal primary antibody (# AQP61-A, 1:250; Alpha Diagnostic International, San Antonio, TX, USA). After three 10 min washes with PBS, coverslips were incubated at room temperature with the fluorescent secondary antibody, rhodamine red X-conjugated affinity pure goat anti-rabbit IgG (H+L) (111-295-045, 1:1000; Jackson ImmunoResearch Europe Ltd., Cambridgeshire, UK), for 1 h. Coverslips were then washed 3 × 10 min with PBS, mounted in ProLong Gold antifade reagent with 4’,6-Diamino-2-Phenylindole (DAPI; Molecular Probes), and examined with a TCS SP5 II confocal microscopy system (Leica Microsystems) equipped with a DM IRBE inverted microscope (Leica Microsystems). Images were acquired with a 60× objective, visualized, and analyzed by LAS AF Lite software (Leica Microsystems Application Suite Advanced Fluorescence Lite version 2.6.0). Negative controls were performed by incubating slices with non-immune serum.

To evaluate the colocalization, 3D images were analyzed with just another Colocalization Plugin (JACoP) from Fiji to obtain the Pearson’s correlation coefficient r, Manders’ colocalization coefficient (M1 and M2), and Van Steensel’s cross-correlation Function (CCF) [35,36,37].

### 2.5. Water Permeability Measurements

Osmotic water permeability of mesothelium and MPM cells was measured using the stopped-flow light scattering method [38]. Briefly, the experiments were carried out at room temperature on a stopped-flow apparatus (RX2000, Applied Photophysics, Leatherhead, UK) with a pneumatic drive accessory (DA.1, Applied Photophysics) coupled with a Varian Cary 50 spectrometer (Varian Australia Pty Ltd., Mulgrave, Australia). The scattered light intensity with a dead time of 6 ms was recorded at a wavelength of 450 nm. The time course of cell swelling caused by exposure to the hypotonic gradient (150 mOsm/L) was measured at the acquisition rate of one reading/0.0125 s. Cells behaved as perfect osmometers, and the gradient determined an osmotic water entry, cell swelling, and decreased light scattering. The initial rate constant of volume changes (k) in cells was obtained by fitting the points of the time course of light scattering with a one-phase exponential decay equation, calculated by computerized least squares regression (GraphPad Prism 4.00, La Jolla, CA, USA, 2003). Recently, it has been demonstrated that the osmotic water permeability of AQPs is indicative of H_2_O_2_ permeability [9]. The water permeability coefficient, P_f_, was calculated as previously described by Wiener et al. [39], from the following equation:

P_f_ = k ∙ V_0_/ΔC ∙ V_W_ ∙ A

where ΔC is the osmotic gradient, V_W_ is the molar water volume, V_0_ is the cell volume, and A is the cell surface area.

To study the effect of oxidative stress on water permeability, MPM cells were divided into five groups: (a) normal untreated cells (control); (b) cells treated for 45 min with 50 μM H_2_O_2_ (exogenous oxidative stress); (c) cells incubated for 3 h at 42 °C (endogenous oxidative stress); (d) stressed cells treated for 15 min with 15 mM β-mercaptoethanol to restore the normal condition; (e) cells pretreated with 10 mM (final concentration) diphenyleneiodonium chloride (DPI) and incubated for 3 h at 42 °C. The effect of mercury chloride, a known aquaporins inhibitor, was evaluated to confirm the AQP involvement in water permeability. For this purpose, three groups were considered: (a) normal untreated cells (control); (b) cells treated for 15 min with 100 μM HgCl_2_; (c) cells treated with HgCl_2_ and then restored with 15 mM β-mercaptoethanol for 15 min. To study the effect of oxidative stress on water permeability of AQP6-silenced cell lines, silenced and not-silenced cells were divided into two groups: (a) normal untreated cells (control) and (b) cells treated for 45 min with 50 μM H_2_O_2_ (exogenous oxidative stress).

### 2.6. Hydrogen Peroxide Influx Measurements

Hydrogen peroxide influx in mesothelium and MPM cells was measured by a fluorescence method using the 5-(and-6)-chloromethyl-2’,7’-dichlorodihydro-fluorescein diacetate and the acetyl ester reagent (CM-H_2_DCFDA) (invitrogen). Briefly, cells were centrifuged at 200 rcf for 5 min. The cell pellet was resuspended in PBS and divided into two groups: (a) normal untreated cells (control) with CM-H_2_DCFDA reagent that was added at 10 µM final concentration and incubated for 1 h at room temperature; and (b) cells incubated for 2 h 45 min at 42 °C (endogenous oxidative stress) with CM-H_2_DCFDA reagent that was added at 10 µM final concentration and incubated for 15 min at 42 °C. Thereafter, cells were centrifuged again, and the pellet was resuspended in PBS, just before the measurement. Cells in different experimental conditions were injected with 50 μM H_2_O_2_ final concentration or distilled water; cellular H_2_O_2_ levels were detected over 15 min with CLARIOs by using a CLARIOstar® microplate reader (BMG LABTECH, Ortenberg, Germany).

### 2.7. Gene Silencing

siRNA targeting AQP6 was purchased by DharmaconTM (ON-TARGETplus Human AQP6 (363) siRNA–SMARTpool, L-011579-00-0005, DharmaconTM, Horizon Discovery Group, Waterbeach, UK). Scrambled siRNA was used as a negative control. siRNA targeting AQP3 and AQP5 were purchased from Merck (Milan, Italy) MISSION esiRNA (human AQP3, EHU071641; human AQP5, EHU046331).

For AQP3 and AQP5 gene silencing, cells were transfected with siRNA oligonucleotides (5 μM) or equimolar scramble siRNA using the N-ter Nanoparticle siRNA Transfection System (N2913, Merck, Milan, Italy). Briefly, once the monolayer cells had reached 50% confluency, the medium was removed and substituted with a fresh medium containing target siRNA nanoparticle formation solution (NFS). siRNA (5 μM final concentration) was diluted in siRNA dilution buffer (N0413) and mixed with N-TER peptide (N2788) pre-diluted in distilled water, according to the manufacturer’s instructions to create the NFS. After 30 min incubation at 37 °C, the NFS was diluted in a medium, added to the cells, and incubated at 37°C for 24 h.

For AQP6 gene silencing, cells were transfected with siRNA oligonucleotides (20 nM) or equimolar scramble siRNA using the INTERFERin siRNA transfection reagent (# 409-10, Polyplus transfection, Illkirch-Graffenstaden, France). Briefly, once the monolayer cells had reached 50% confluency, the medium was removed and substituted with a fresh medium containing the silencing solution. siRNA (20 nM final concentration) was diluted in Opti-MEM and then mixed with INTERFERin siRNA transfection reagent, according to the manufacturer’s instructions to create the silencing solution. After 15 min incubation at room temperature, the silencing solution was diluted in a fresh medium, added to the cells, and incubated at 37 °C for 48 h.

The effectiveness of silencing was determined by immunoblotting, and silenced cells were used after 24 h (AQP3- and AQP5-silenced cells) and 48 h (AQP6-silenced cells) from transfection.

### 2.8. Hyper7-NES Transfection

The plasmid for the mammalian expression of cytoplasm targeted ultrasensitive hydrogen peroxide indicator HyPer7 for optical imaging (pCS2+HyPer7-NES) was a generous gift from Vsevolod Belousov (IBCh, Moscow, Russia) (Addgene plasmid # 136467; http://n2t.net/addgene:136467 (accessed on 9 June 2022; RRID: Addgene_136467) [40]. Mesothelial and MPM cells were seeded into 2 mL plastic dishes, and HyPer7-NES transfection (3 μg DNA / dish) was performed when cells reached the 60–70% confluency using the JetOPTIMUS DNA transfection reagent (# 117-15, Polyplus transfection, Illkirch-Graffenstaden, France) according to the manufacturer’s instructions. Briefly, the medium was removed and substituted with Opti-MEM containing the plasmid DNA and the transfection reagent. Plasmid DNA (3 μg) was diluted in JetOPTIMUS Buffer (# 717-60, Polyplus transfection, Illkirch-Graffenstaden, France) and then mixed with JetOPTIMUS reagent according to the suggested ratio 1:1 between μg of DNA and μL of transfection reagent. After 10 min incubation at room temperature, the solution containing the DNA was added drop-wise to the cells and incubated at 37 °C for 4 h. Then, the media were exchanged for fresh culture media. All the experiments were performed 24 h after transfection.

### 2.9. Intracellular H_2_O_2_ Detection by HyPer7-NES Imaging

To reveal the intracellular changes in H_2_O_2_, Hyper7 oxidation should be measured using a ratiometric method [40]. Confocal images were collected every 1–2 s for 1 to 5 min by dual excitation at 420 nm and 490 nm, and the emission was collected at 530 nm. Preliminary experiments showed that results obtained by ratiometric measurements were similar to those obtained by measuring the fluorescence of the HyPer7 biosensor excited at 490 nm and the emission collected at 530 nm. For this reason, the following method was routinely used. An Olympus BX41 microscope with a 60× water immersion objective (LUMPlanFI 60× / 0.90 w, Olympus) was used to visualize the fluorescence of transfected cells. Cells expressing HyPer7-NES were washed with a physiological buffer (140 mM NaCl, 5 mM KCl, 2 mM CaCl_2_, 1 mM MgCl_2_, 10 mM D-glucose, and 1 mM HEPES, pH 7.4) and incubated for 10 min at room temperature with the same buffer. For the fluorescence imaging of live cells, the HyPer7-NES biosensor was excited at 490 nm and emission was collected at 530 nm. Images were acquired using a CCD camera (DMK 33UP1300) and collected at 10 fps by IC capture software. H_2_O_2_ was added to the cells at a final concentration of 50 μM. Image processing was performed with Image J.

### 2.10. MTT Assay

The proliferative capacity of the three cell lines during AQP6 silencing was evaluated with 3-(4, 5-dimethylthiazole-2-yl)-2,5-diphenyl tetrazolium bromide (MTT assay). MTT was performed after AQP6 silencing (24 h, 48 h, and 72 h after silencing). Briefly, cells were seeded at 50% confluency (25,000 cells/well) in duplicate in 12-well plates. The next day, the medium was substituted with 0.5 mg/mL MTT solution in PBS and incubated for 4h at 37 °C in a humidified atmosphere of 95% air with 5% CO_2_. During the incubation, the MTT is reduced into formazan crystals by viable cells. After the incubation, the MTT solution was replaced by isopropanol with 0.01% 1M HCl to solubilize the formazan products. Absorbance values were read at 570 nm with Cary 50 UV spectrophotometer (Agilent Technologies, Cernusco sul Naviglio (MI), Italy).

The proliferative capacity of the three cell lines after AQP6 silencing was also evaluated under oxidative stress conditions by H_2_O_2_ treatment. The AQP6 silencing was performed, as indicated above (see Section 2.7. *Gene Silencing*). Cells were treated with 400 µM H_2_O_2_ final concentration after 3 h from silencing. The next day, cell culture medium was substituted with fresh medium added with 800 µM H_2_O_2_, i.e., the final concentration. After 2 h, the medium was substituted and AQP6 gene silencing was performed again to enhance the silencing stability. The MTT assay was performed after 48 h from the first silencing.

### 2.11. Protein Content

The protein content was determined with the Bradford method [41], using bovine serum albumin as standard.

### 2.12. Statistics

All data were expressed as means ± standard error mean (SEM) or standard deviation (SD). The significance of the differences of the means was evaluated by using one-way ANOVA, followed by Newman–Keuls’s *Q* test or Student’s *t*-test. All statistical tests were carried out with GraphPad Prism 4.00, 2003.

## 3. Results

### 3.1. Aquaporins-3, -5, -6, -9, and -11 Are Expressed in MeT-5A, REN, MSTO-211H Cell Lines

**Figure 1:**
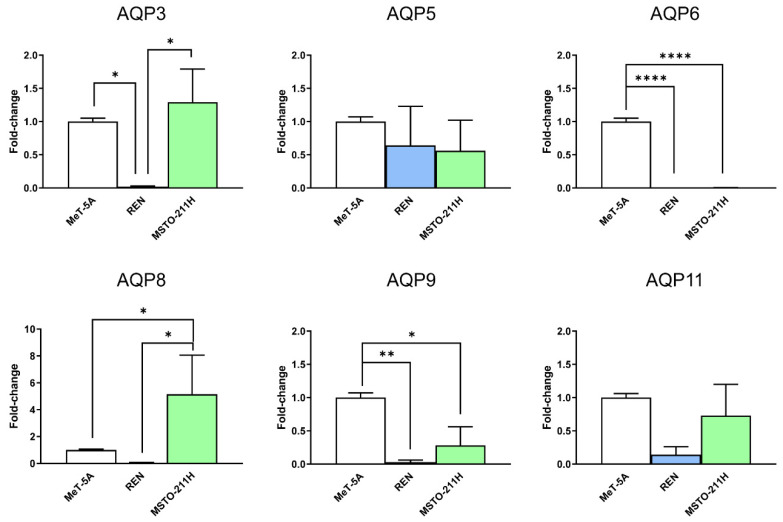
qRT-PCR reaction analysis of AQP3, AQP5, AQP6, AQP8, AQP9, and AQP11 expression in mesothelial (MeT-5A, in white), epithelioid, and biphasic MPM cell lines (REN and MSTO-211H, in blue and green, respectively). Bars represent the mean ± SEM of fold change values (n = 4). *, *p* < 0.05; **, *p* < 0.01; ****, *p* < 0.0001 (ANOVA followed by Newman–Keuls’s *Q* test).

First, using qRT-PCR, we explored the expression in mesothelial (MeT-5A), epithelioid, and biphasic MPM (REN, MSTO-211H) cell lines of the main peroxiporins, and of AQP6 whose expression was hypothesized after preliminary functional experiments. AQP3, 5, 6, 8, 9, and 11 mRNA were expressed in the three cell lines. Agarose gel electrophoresis of qPCR reaction products revealed single bands of the expected size: 147 bp for AQP3, 142 bp for AQP5, 103 bp for AQP6, 106 bp for AQP8, 133 bp for AQP9, and 115 bp for AQP11 (not shown). qRT-PCR showed that AQP3 mRNA is downregulated in REN, AQP6, and AQP9 in both MPM cell lines, and AQP8 was upregulated in MSTO-211H (Figure 1).

**Figure 2:**
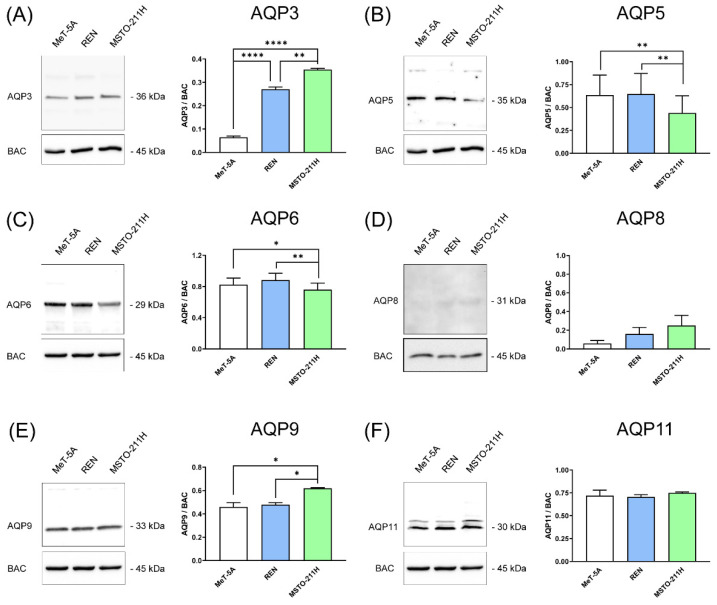
Immunoblotting and densitometric analysis of AQP3 (**A**), AQP5 (**B**), AQP6 (**C**), AQP8 (**D**), AQP9 (**E**), and AQP11 (**F**) in mesothelial (MeT-5A, in white), epithelioid, and biphasic MPM cell lines (REN and MSTO-211H, in blue and green, respectively). (**Left panels**) Representative blots of three different experiments are shown. Lanes were loaded with 30 μg of proteins, probed with affinity-purified antibodies, and processed as described in Materials and Methods. The same blots were stripped and re-probed with anti-β-actin (BAC) antibody, as housekeeping. Major bands of the expected molecular weights are shown. (**Right panels**) Densitometry of AQP protein levels in the three cell lines. Each bar represents the mean ± SEM of the normalized values of AQP protein expression. *, *p* < 0.05; **, *p* < 0.01; ****, *p* < 0.0001. (ANOVA followed by Newman–Keuls’s *Q* test).

The total membrane preparations from MeT-5A, REN, and MSTO-211H were analyzed by immunoblotting using affinity-purified antibodies. All AQP proteins were detected except AQP8 (whose expression was nil or negligible) (Figure 2). The lack of a relevant quantity of AQP8 was also demonstrated by immunocytochemistry experiments (Figure S1).

Immunoblots showed major bands with sizes compatible with those reported in the literature. Densitometry showed that AQP3 protein expression was statistically upregulated in MPM cell lines, AQP5 and AQP6 protein expression was downregulated in MSTO-211H, while AQP9 was upregulated in MSTO-211H by about 35 percent (Figure 2). The expression of AQP11 protein did not change in the three cell lines (Figure 2).

### 3.2. Aquaporin-3, -5, -6 Localization in MeT-5A, REN, MSTO-211H Cell Lines

**Figure 3:**
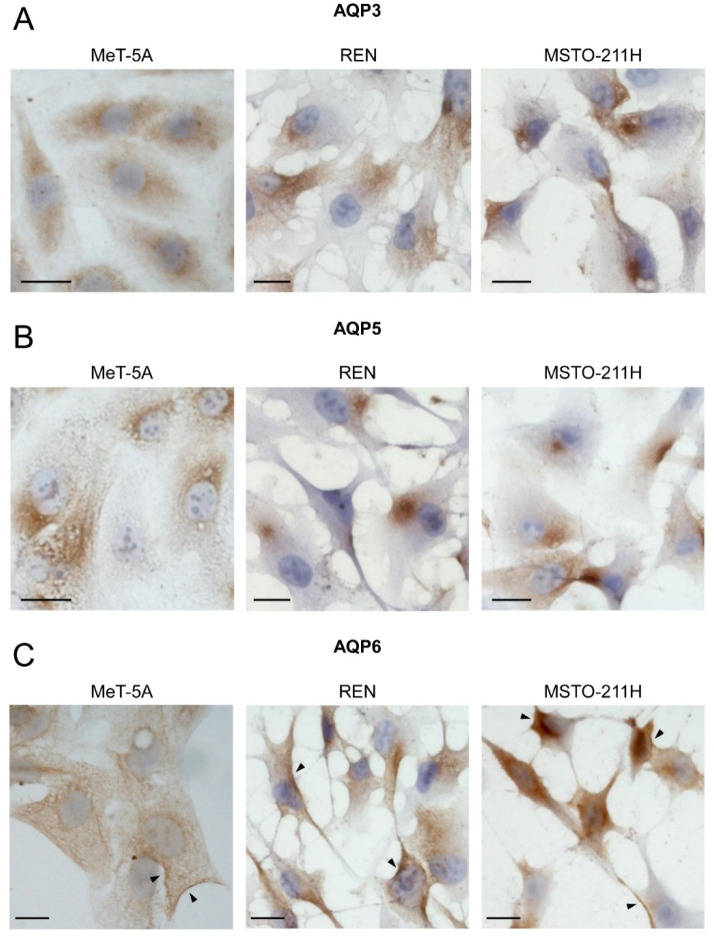
Immunocytochemical localization of AQP3 (**A**), AQP5 (**B**), and AQP6 (**C**) proteins in MeT-5A, REN, and MSTO-211H cell lines. AQP3 and AQP5 staining appeared to be confined mainly to intracellular structures. AQP6 staining in MeT-5A is mainly intracellular and has a low expression in the plasma membrane. REN and MSTO-211H cell lines showed strong labelling in discrete areas of the plasma membrane, in addition to the intracellular expression. Arrowheads indicate the localization in plasma membranes. Scale bar: 20 μm.

In this study, the role of AQP3, AQP5, and AQP6 is examined. The cellular localization of AQP3, AQP5, and AQP6 in mesothelial and MPM cell lines was first studied by immunocytochemistry. As shown in Figure 3, the anti-AQP3 and anti-AQP5 antibodies labeled only intracellular structures (Figure 3A,B), while the anti-AQP6 antibody, in addition to intracellular staining, showed strong labeling in discrete areas of the plasma membrane of MPM cell lines (Figure 3C). The negative controls incubated with non-immune serum showed an absence or negligible signal in the three cell lines (Figure S2).

**Figure 4:**
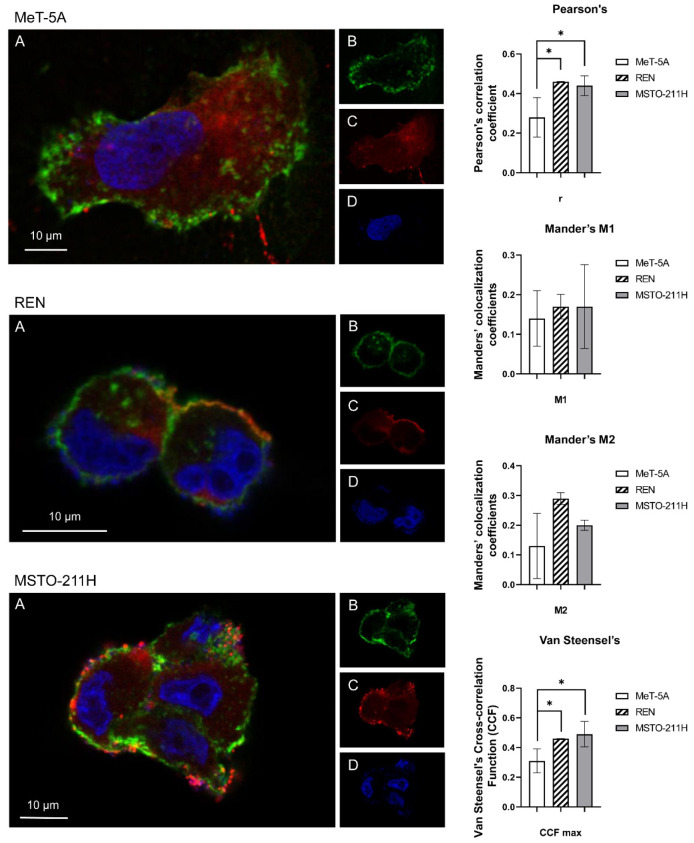
Representative images of confocal laser scanning microscopy and 3D colocalization analysis of AQP6 (**A–D**) with concanavalin A (ConA) in MeT-5A, REN, and MSTO-211H cell lines. Green labeling indicates the presence of ConA (**B**), red labeling indicates the expression of AQP6 (**C**), while DAPI (blue, **D**) indicates counterstained nuclei. Yellow labelling shows the colocalization signal of AQP with ConA (**A**). Scale bar: 10 μm (right panel). Statistical analysis of Pearson’s correlation coefficient r, Manders’ colocalization coefficient (M1 and M2), and Van Steensel’s maxima cross-correlation function (CCF) were obtained from 4 different double immunofluorescence experiments with anti-AQP6 antibody and anti-AQP6 antibodies. Coefficients were determined by 3D analysis of at least 20 cells for each cell line (8–15 z-stack for image) using the JACoP plugin of Fiji. The columns represent the mean ± SD of the coefficient values; *, *p* < 0.05 (ANOVA, followed by Newman–Keuls’s *Q* test).

Colocalization experiments were performed to gain more evidence about the localization of AQP6 on the plasma membrane (Figure 4). Green labeling indicates the presence of ConA (Figure 4A,B), red labeling indicates the expression of AQP6 (Figure 4A,C), while DAPI (blue) indicates counterstained nuclei (Figure 4A,D). Double-label immunofluorescence showed that AQP6 and ConA, as well as leptin that recognizes the plasma membrane, colocalized, as per the resulting yellow fluorescence in merged images of REN and MSTO-211H cell lines (Figure 4A).

In contrast, in MeT-5A cells, the colocalization of AQP6 and ConA (Figure 4A) is not so evident, as yellow fluorescence is not noticeable in the merged image (Figure 4A). Control experiments were also conducted by substituting the antibodies with non-immune serum in the MSTO-211H cell line and did not show any labeling (Figure S3).

To evaluate the possible colocalization of AQP6 with ConA, we analyzed 3D images using JACoP from Fiji and quantified the Pearson’s correlation coefficient r, Manders’ colocalization coefficients (M1 and M2), and Van Steensel’s cross-correlation function (CCF) (Figure 4, right panel). Pearson’s correlation coefficients were included between0.5 to 0.5, so no conclusions can be drawn [35]. Moreover, Pearson’s correlation coefficient r in MeT-5A was significantly lower than that of REN and MSTO-211H. Manders’ overlap coefficient M1 and M2 indicate the percentage of the ConA (green) signal coincident with an AQP6 signal (red channel), respectively, over its total intensity and vice versa [36]. The results show that the Manders’ coefficients did not differ significantly in the three cell lines. Cross-correlation analysis [37] showed that CCF maxima values in MeT-5A were significantly lower than those of REN and MSTO-211H.

Taken together, the results suggest that AQP6 is not expressed throughout the plasma membrane but only in discrete areas of it. Furthermore, the AQP6 expression in the plasma membrane is significantly lower in MeT-5A than in REN and MSTO-211H.

### 3.3. Effect of Oxidative Stress and Mercury Chloride on Water Permeability of MeT-5A, REN, MSTO-211H Cell Lines

First, we studied the effect of oxidative stress and mercury chloride on the osmotic permeability to water of AQPs, since the latter is indicative of the permeability to H_2_O_2_, as previously demonstrated. The osmotic water permeability was measured by exposing the cells to a hypotonic medium. This causes rapid cell swelling and a decreased scattered light intensity (Figure S4). The initial rate constant k was obtained by curve fitting with a one-phase exponential decay equation.

By comparing the osmotic permeability of the three cell lines by calculating the osmotic permeability coefficient P_f_ as indicated in Materials and Methods, we found that MeT-5A cells have a significantly higher P_f_ as compared to REN and MSTO-211H cells [4.88 × 10^−2^ ± 1.60 × 10^−3^ cm/s (n = 24) versus 2.87 × 10^−2^ ± 7.31 × 10^−4^ cm/s (n = 25) and 3.23 × 10 ^−2^ ± 5.48 × 10^−4^ cm/s (n = 24)].

**Figure 5:**
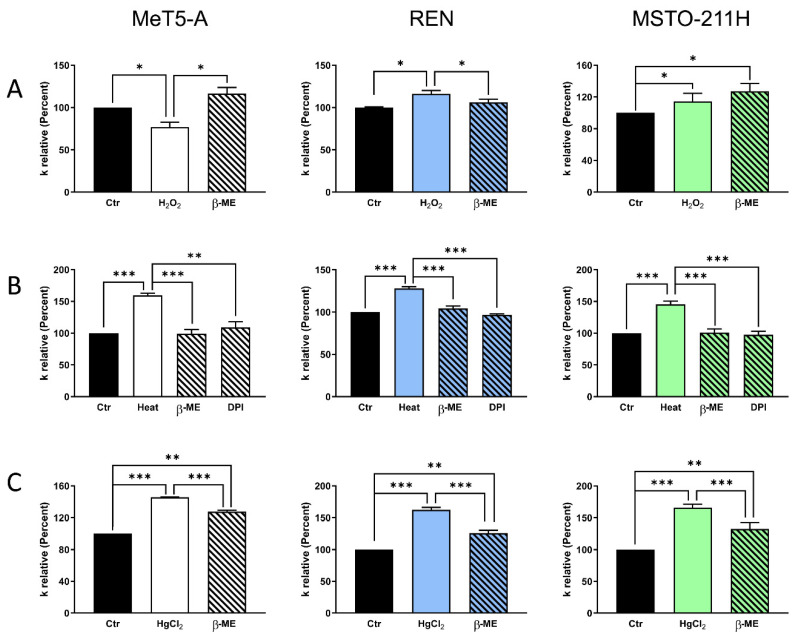
Effect of oxidative stress (**A**,**B**) and mercury (**C**) on the water permeability of mesothelial cells (MeT-5A), epithelioid (REN), and biphasic (MSTO-211H) MPM cells. (**A**) Cells were exposed to 150 mOsm osmotic gradients in three different conditions: untreated cells (Ctr), cells treated with H_2_O_2_ (H_2_O_2_), and cells treated with β-mercaptoethanol after H_2_O_2_ treatment (β-ME). (**B**) Cells were exposed to 150 mOsm osmotic gradients in four different conditions: untreated cells (Ctr), heat-stressed cells (Heat), cells treated with β-mercaptoethanol after heat stress (β-ME), and cells treated with diphenyleneiodonium chloride (DPI) before heat stress (DPI). (**C**) Cells were exposed to 150 mOsm osmotic gradients in three different conditions: untreated cells (Ctr), cells treated with mercury chloride (HgCl_2_), and cells treated with β-mercaptoethanol after HgCl_2_ treatment (β-ME). *, *p* < 0.05; **, *p* < 0.01; ***, *p* < 0.001. Bars represent the osmotic water permeability of MeT-5A cells expressed as a percent of k relative. Values are means ±SEM of 4–15 single shots for each of 4 different experiments (ANOVA, followed by Newman–Keuls’s *Q* test).

Mesothelial cells (MeT-5A) treated with H_2_O_2_ showed a significant water permeability reduction, which was restored by subsequent β-mercaptoethanol (β-ME) treatment (Figure 5A). On the contrary, the cells subjected to heat stress did not show a reduction in water permeability, but rather showed an increase of about 55% (Figure 5B). The enhanced AQP permeability in heat-stressed cells was also reversed by β-ME post-treatment, and by pre-treating the cells with DPI, a potent inhibitor of NADPH oxidase and iNOS/eNOS (Figure 5B). The involvement of AQPs in cell swelling was studied by pretreating the cells with mercury chloride, a well-known AQP inhibitor [42,43]. Surprisingly, the results show an increase in water permeability (45%; *p* < 0.05, ANOVA followed by Newman–Keuls’s *Q* test) (Figure 5C), probably due to the presence of plasma membrane AQP6 [44]. The subsequent treatment with β-ME partially restored the water permeability values (Figure 5C).

As for the epithelioid and biphasic MPM cell lines, REN and MSTO-211H, the H_2_O_2_ treatment increased the water permeability of the first, but did not change that of the latter (Figure 5A). The heat stress treatment increased the water permeability of REN and MSTO-211H by about 20% and 60%, respectively (Figure 5B). The post-treatment with β-ME and the pre-treatment with DPI prevented the enhancement of AQP permeability by heat stress (Figure 5B). The mercury treatment increases water permeability by 62% for epithelioid MPM cells and 66% for biphasic MPM cells, and the post-treatment with β-ME partially restored the water permeability (*p* < 0.05, ANOVA followed by Newman–Keuls’s *Q* test) (Figure 5C).

### 3.4. Hydrogen Peroxide Permeability of MeT-5A, REN, MSTO-211H Cell Lines

**Figure 6:**
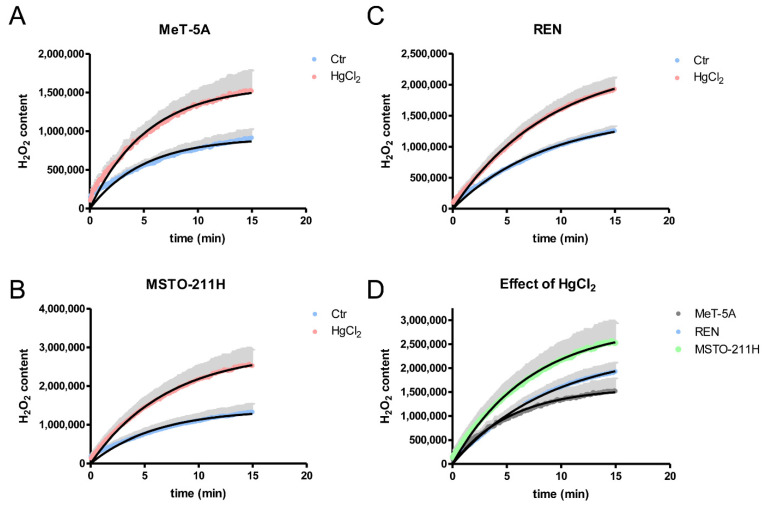
Effect of mercury chloride (HgCl_2_) treatment on the hydrogen peroxide permeability of mesothelial (MeT-5A; **A**), epithelioid (REN; **B**), and biphasic (MSTO-211H; **C**) MPM cell lines. (**D**) shows the compared effect of HgCl_2_ on hydrogen peroxide permeability in three cell lines. Hydrogen peroxide permeability was measured by loading the cells with CM-H_2_DCFDA reagent as described in Materials and Methods. Curves represent the time course of H_2_O_2_ transport, expressed as H_2_O_2_ content. Values are mean ± SEM (in gray) of three-time courses for each of four different experiments. Ctr, controls (**A–C**) *p* < 0.0001 (Student’s *t*-test). (**D**) Y_max_ values obtained by one-phase exponential association were (Mean ± SEM): Met-5A, 1.59 × 10^6^ ± 2.3 × 10^4^; REN, 2.39 × 10^6^ ± 4.2 × 10^4^; MSTO-211H, 2.90 × 10^6^ ± 6.4 × 10^4^. The values were statistically different with MSTO-211H > REN > Met-5A (*p* < 0.05; ANOVA, followed by Newman–Keuls’s *Q* test).

The direct involvement of AQPs in the H_2_O_2_ permeability of mesothelial and MPM cell lines was measured by a fluorescence method using the CM-H_2_DCFDA reagent. Two different experimental conditions were used: untreated cells (Ctr) and cells treated with 100 μM HgCl_2_. Three types of cells were able to transport H_2_O_2_, as shown by the increased H_2_O_2_ accumulation after its addition to the extracellular medium (Figure 6A–C). Treatment with HgCl_2_ did not decrease the permeability to H_2_O_2_, but instead increased it by about 66% in MeT-5A. 53% in REN and 90% in MSTO-211H cells (Figure 6A–D), further confirming the presence of AQP6. In panel D, the permeabilities to H_2_O_2_ after treatment with HgCl_2_ in the three cell lines were compared, and the values of Y_max_ were statistically higher in MSTO-211H > REN > Met-5A.

### 3.5. Effect of Oxidative Stress on Water Permeability of Me, T-5A, REN, and MSTO-211H Cell Lines with Reduced AQP6 Expression

The increase in water and H_2_O_2_ permeability after mercury treatment suggested the involvement of AQP6 in redox modulation in mesothelial and even more in MPM cells. AQP6 silencing was performed with either a siRNA selectively targeting or a scrambled (i.e., control) siRNA (see Materials and Methods) to further demonstrate it. The effectiveness of silencing was determined by immunoblotting and the KO-cells were used 48 h after transfection (Figure S5). Immunoblot analysis revealed that the content of AQP6 protein decreased by approximately 70% in MeT-5A cells, 62% in REN cells, and 44% in MSTO-211H 48 h after siRNA transfection (Figure S5). 

**Figure 7:**
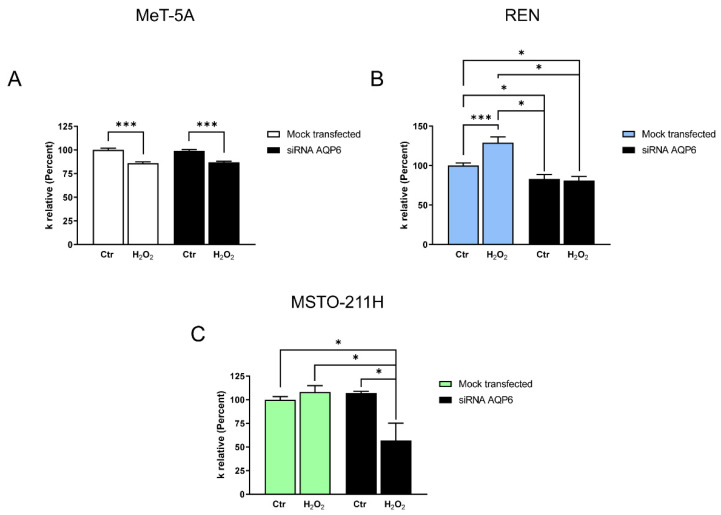
Effect of oxidative stress on water permeability of MeT-5A (**A**), REN (**B**), and MSTO-211H (**C**) cell lines after AQP6 gene silencing. Mock-transfected (scrambled, mock-transfected) and silenced cells (siRNA AQP6) were exposed to 150 mOsm osmotic gradients in two different conditions: untreated cells (Ctr) and cells treated with H_2_O_2_ (H_2_O_2_). *, *p* < 0.05; ***, *p* < 0.001. Bars represent the osmotic water permeability of cells expressed as a percent of k relative. Values are means ± SEM of 4–15 single shots for each of 4 different experiments (ANOVA, followed by Newman–Keuls’s *Q* test).

Consistent with this finding, the genetic suppression of AQP6 reduced the water permeability of REN compared with “control” REN cells (mock-transfected) (Figure 7). On the contrary, both MeT-5A and MSTO-211H cells were unaffected by AQP6 silencing (Figure 7). This result can be easily explained for mesothelial MeT-5A cells because immunolocalization experiments showed a very limited amount of functional AQP6 in the plasma membrane. As for MSTO-211H cells, the unaffected water permeability after AQP6 silencing could be explained by an upregulation of other AQPs. To prove it, the expression of the AQPs in MSTO-211H cells silenced for AQP6 was investigated. As shown in Figure S6, AQP9 transcript expression was upregulated in AQP6-null cells as compared to silenced mock cells.

To further demonstrate the importance of AQP6 and the specificity of the phenomenon, the intracellular AQPs, i.e., AQP3 and AQP5, were silenced, and water permeability was measured. The silencing of both AQP3 and AQP5 did not affect the water permeability in the three cell lines (Figure S7).

AQP6-null MeT-5A cells treated with H_2_O_2_ showed the same behavior as control mock-transfected cells demonstrating a reduced water permeability after H_2_O_2_ treatment (Figure 7A). Conversely, REN and MSTO-211H cells with reduced AQP6 showed a different sensitivity of the water permeability to H_2_O_2_ treatment compared to mock-transfected cells (Figure 7B,C). In REN-silenced cells, the water permeability of treated and untreated H_2_O_2_ cells was similar and differed from mock cells in which H_2_O_2_ treatment increased permeability (*p* < 0.05, ANOVA followed by Newman–Keuls’s *Q* test) (Figure 7B). Similarly, in MSTO-211H silenced for AQP6, the water permeability was reduced by H_2_O_2_ treatment, while in control cells the treatment with H_2_O_2_ was ineffective (Figure 7C). This result supports the involvement of AQP6 in the increased water permeability following H_2_O_2_ treatment of MPM cells.

### 3.6. Hydrogen Peroxide Permeability of MeT-5A, REN, MSTO-211H Cell Lines with Reduced AQP6 Expression

**Figure 8:**
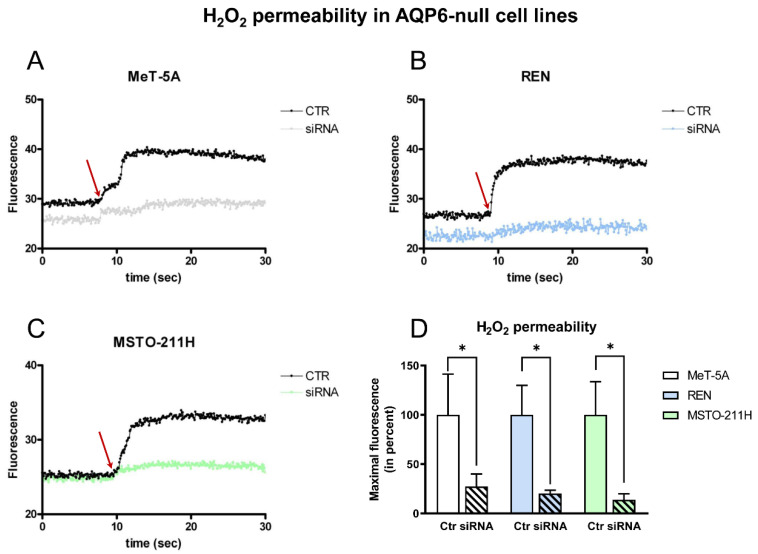
Effect of aquaporin-6 (AQP6) silencing on the H_2_O_2_ permeability MeT-5A (**A**), REN (**B**), and MSTO-211H (**C**) cell lines. (**A**–**C**) HeLa cells were silenced with AQP6 siRNA and then transiently transfected with HyPer7 sensor, as described in Materials and Methods. Control (scrambled; CTR) and silenced cells (siRNA) were exposed to 50 μM H_2_O_2_ gradient (final concentration). Curves show the time course of H_2_O_2_ transported into the cells after H_2_O_2_ injection (red arrow). (**D**) Bars represent the H_2_O_2_ permeability of cells expressed as a percent of maximal fluorescence. Values are means ± SEM of cells for each of 3 different experiments in triplicates. *, *p* = 0.0414, *p* = 0.0260, *p* = 0.0275 versus Ctr, for Met-5A, REN, and MSTO-211H, respectively (Brown–Forsythe and Welch ANOVA tests).

The direct involvement of AQP6 in the permeability of H_2_O_2_ and thus in the cellular redox control was then demonstrated using the H_2_O_2_-sensitive probe HyPer7-NES to efficiently measure the H_2_O_2_ changes in the cytosolic compartment [40]. The addition of 50 μM H_2_O_2_ (final concentration), indicated in the figure with a red arrow, clearly showed an increase in the emission fluorescence of the Hyper7 sensor in the MeT-5A, REN, and MSTO-211H cell lines (Figure 8A–C). In the three cell lines, AQP6 silencing greatly reduced the transport of H_2_O_2_ into the cytosol compared with mock-transfected cells. Figure 8D shows that the maximal fluorescence, obtained by curve fitting with a one-phase exponential association equation of H_2_O_2_ transport in AQP6-silenced cells, was reduced by about 84% compared with control mock cells. The experiment confirms that the cells efficiently conduit H_2_O_2_, and the suggested involvement of AQP6.

### 3.7. MeT-5A, REN, and MSTO-211H Cells Proliferation after AQP6 Gene Silencing

**Figure 9:**
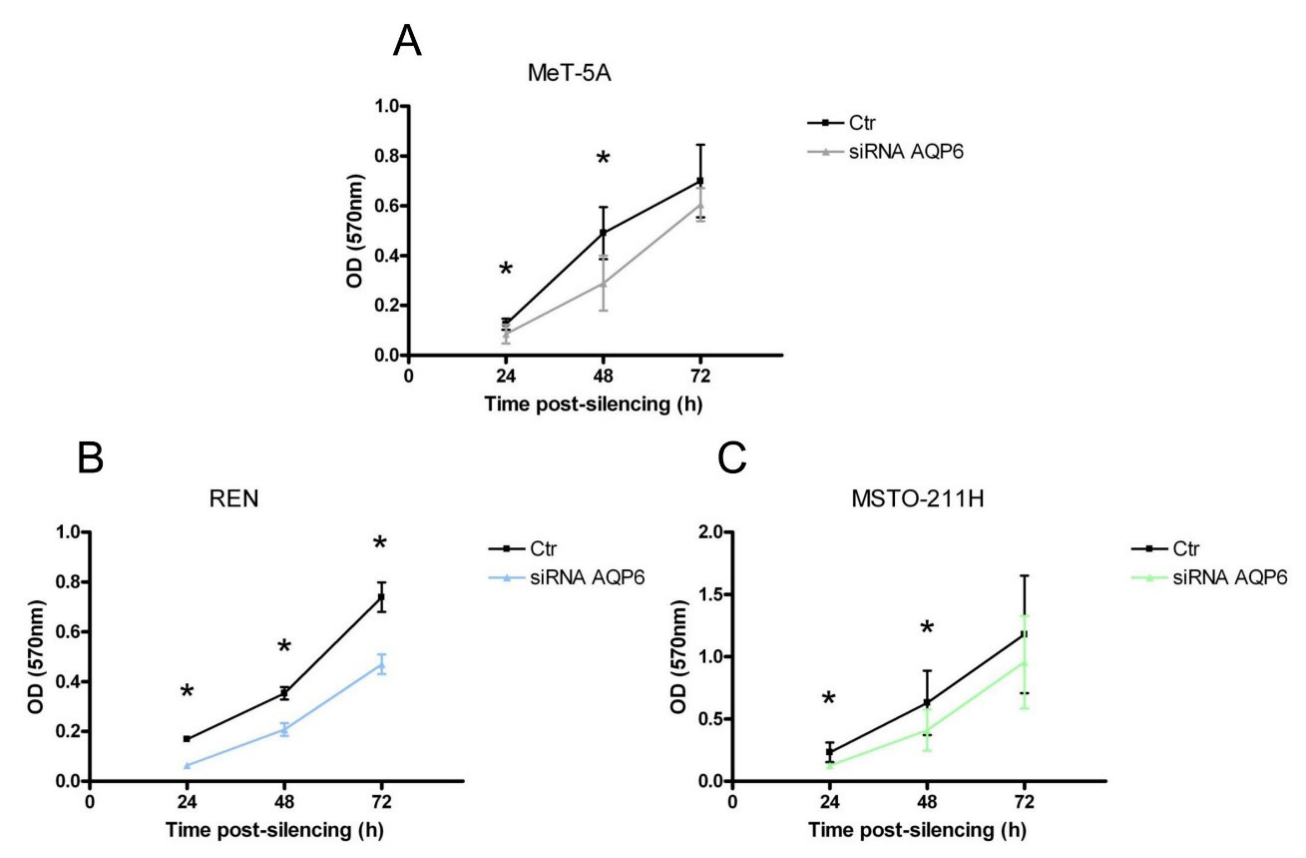
Effect of aquaporin-6 (AQP6) silencing on the cell proliferation of MeT-5A (**A**), REN (**B**), and MSTO-211H (**C**) cultured cells. Cell growth was evaluated by measuring the OD at 570 nm at 24, 48, and 72 h after cell silencing compared with scrambled silenced control cells. The initial cell number was about 25,000 cells/well. AQP6-null cells (siRNA AQP6) showed a significantly decreased proliferation compared with controls after 24 and 48 h from silencing in Met-5A and MSTO-211H cells and after 24, 48, and 72 h from silencing in REN cells. Values (OD at 570 nm normalized to the cell number) are mean ± SEM of cells for each of 4 different experiments. *, *p* < 0.05 (Student’s *t*-test).

The cell proliferation levels in the AQP6 siRNA-transfected MeT-5A, REN, and MSTO-211H cells, compared with mock-transfected control cells, were measured using an MTT proliferation assay at 24, 48, and 72 h post-transfection. Results show that the number of viable cells in the AQP6-siRNA group of MeT-5A, REN, and MSTO-211H cells was significantly lower compared with that of the scrambled siRNA group 24, 48 h (and 72 h in REN) after AQP6-siRNA transfection (*p* = 0.012, Student’s *t-test*) (Figure 9).

**Figure 10:**
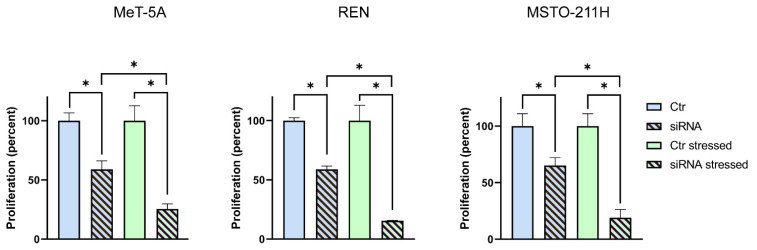
Effect of oxidative stress on cell proliferation of MeT-5A, REN, and MSTO-211H cells silenced for aquaporin-6 (siRNA) and mock-transfected (Ctr). Cell growth was evaluated by measuring the OD at 570 nm at 48 h after cell silencing compared with mock-transfected control cells. Oxidative stress was produced in siRNA and mock-transfected cells, as indicated in Materials and Methods. Values (expressed as the percent of proliferation) are means ± SEM of cells for each of 4 different experiments. *, *p* < 0.05 (ANOVA, followed by Newman–Keuls’s *Q* test).

Finally, the cell proliferation levels in the AQP6 siRNA-transfected MeT-5A, REN, and MSTO-211H cells were measured under oxidative stress conditions 48 h after the silencing. As shown in Figure 10, AQP6 silencing decreased cell proliferation, but in oxidative stress conditions, cell proliferation was further and dramatically affected; thus, REN and MSTO-211H cells accounted for about 15 and 19% compared to controls, respectively.

## 4. Discussion

The involvement of aquaporin-6 in increasing the resistance to oxidative stress of malignant pleural mesothelioma cells undoubtedly provides new prominent information in understanding the role of peroxiporins in the redox regulation of malignant cells. Moreover, the results suggest a new target in the treatment of this aggressive cancer.

MPM is a malignant tumor of the pleural surface and it is associated with previous asbestos exposure, which often occurs 40 years previously. The prognosis is poor and the median survival ranges from 8 to 14 months from diagnosis [45]. The standard therapeutic strategies for each MPM sub-types (epithelioid, sarcomatoid, and biphasic) are radiotherapy and chemotherapy, even if the results are discouraging.

The ROS and, in particular, hydrogen peroxide (the most abundant) exert the main role in cancer biology [46]. Their elevated concentrations are fundamental in cancer development, cell growth, proliferation, migration, metastasis, and cell survival, which drive genetic instability and DNA damage. More elevated ROS levels may have an anti-tumorigenic effect by inducing ferroptosis. Cancer cells establish a redox balance to maintain high pro-tumorigenic ROS levels, but prevent the anti-tumorigenic (excessive) ROS accumulation and develop resistance to ferroptosis [47]. Among the antioxidant mechanisms, the AQPs may have a leading role in mediating the membrane transport of H_2_O_2_ and regulating its intracellular concentration [8].

Most studies demonstrate that AQPs played leading roles in proliferation, migration, and angiogenesis, necessary for tumor progression, invasion, and metastasis [48], and these reasons were considered promising therapeutic targets for the treatment of tumors.

Only a few studies have investigated the role of AQPs in MPM. High levels of AQP1 expression in MPM tumor cells were found to predict an increase in the survival rate [29,49]. In addition, AQP1 plays a role in the equilibration of the osmotic gradient in pleural effusions, as well as the proliferation, movement, and anchorage-independent growth in MPM [27]. Unfortunately, there are no data on the presence of other AQPs in MPM nor on their function as peroxiporins.

All the AQPs tested were expressed at mRNA and protein levels in MeT-5A, REN, and MSTO-211H cell lines, except AQP8 which was only found at the mRNA level. In addition to the peroxiporins AQP3, AQP5, AQP9, and AQP11, we studied AQP6 expression because preliminary functional results evidenced increased water permeability following treatment with mercury, consistent with the AQP6 presence (Figure 5C) [44,50]. In this study, we focused on the role of AQP3 and AQP5 because most of the studies demonstrated that these AQPs were significantly increased and played prominent roles in the proliferation, migration, and angiogenesis of cancer [48,51,52]. In addition, AQP6 function was also examined because its significant upregulation in stromal tumor tissue was reported [53], but our results show a reduced expression in REN and MSTO-211H compared with MeT-5A.

More relevant were the immunolocalization results. Unexpectedly, AQP3 and AQP5 were expressed in the cytoplasm in the three cell lines, while the immunolabelling signal of anti-AQP6 showed strong labeling in discrete areas of the plasma membrane of MPM cell lines (Figure 3 and Figure 4). The localization in the plasma membrane of an AQP typically located in intracellular structures [50] may suggest the involvement of AQP6 in cancer growth and progression.

Functionally, the osmotic water permeability of mesothelial MeT-5A cells was measured and compared to that of MPM cells. The P_f_ of REN and MSTO-211H was significantly lower than that of MeT-5A (see Results). Katkova et al. [54] reported similar results with a significantly lower Pf in epithelioid (M14K) and sarcomatoid (ZL34) cells compared to that of benign human mesothelial cells Met-5A. Experiments were also performed to evaluate the effect of oxidative stress on water and H_2_O_2_ permeability of mesothelial and MPM cells. ROS are important tumor-inducing, as well as tumor-suppressing, factors [55]. Different mechanisms significantly increase ROS levels in cancer cells, and ROS can influence tumor development by mediating initiation, progression, and metastasis. Most cancer chemotherapeutic agents act by increasing the already high level of intracellular ROS above a critical threshold, leading to cell death mostly via ferroptosis [56,57].

In our experiments, oxidative stress was achieved via cellular incubation with H_2_O_2_ (exogenous stress) and via heat stress (endogenous stress). Contrary to what was observed in MeT-5A mesothelium cells, the water permeability of MPM cells was not reduced by H_2_O_2_ treatment, but it was considerably increased by heat stress (Figure 5). These data suggest the resistance of MPM cells to endogenous oxidative stress and ferroptosis, as these cells are characterized by an increased H_2_O_2_ efflux via AQPs, setting them free from the high levels of H_2_O_2_.

A recent study by Soveral’s group showed that AQP5 gating could be involved in the fine-tuning of cell sensitivity and/or resistance to oxidative external conditions and could induce cell proliferation and migration [58].

The presence of a functioning AQP6 in the plasma membrane of the three types of cells was further demonstrated by measuring the water and H_2_O_2_ permeability after the treatment with mercury chloride and by measuring the water permeability in MPM cells resuspended in acid buffer (Figure S8). Figure 5, Figure 6 and Figure S8 show increased water permeability, consistent with the AQP6 expression [44,50], but also enhanced H_2_O_2_ permeability. This demonstrates, for the first time, that AQP6 possesses H_2_O_2_ permeability.

To clarify the contribution of AQP3, 5, and 6 to the overall water and H_2_O_2_ permeability of Met-5A, REN, and MSTO-211H, as well as their response to oxidative stress, we performed gene silencing followed by functional experiments. AQP3 and AQP5 silencing did not modify the water permeability of the cells, further demonstrating their intracellular expression (Figure S7). These results confirmed previous observations that AQP3 and AQP5 (and AQP1) were expressed in MPM, but do not have any effect on the prognostic parameters [59]. AQP6 silencing reduced the water permeability of REN, but not of Met-5A and MSTO-211H. While the result in MeT-5A can be explained by a limited AQP6 localization in the plasma membrane, in MSTO-211H, the overall water permeability was unaffected for the compensatory upregulation of AQP9 (Figure S6). However, both MPM cells silenced for AQP6 did not show the increased permeability following H_2_O_2_ treatment compared to mock control cells (Figure 7B,C).

To evaluate whether AQP6 can act as a peroxiporin, MeT-5A, REN, and MSTO-211H were engineered to express the HyPer7-NES sensor in the cytosol. AQP6 silencing showed that HyPer7 oxidation was severely inhibited (about 84% reduction) compared with mock-transfected cells (Figure 8).

As a whole, MPM cells have a higher amount of AQP6 resident in the plasma membrane compared to control cells that prevent oxidative stress onset and ferroptosis. The increased expression of AQP6 in the membrane may be caused by the high oxidative state of the tumor cells, similar to what was previously demonstrated for AQP4 in astrocytes [60]. The authors found that oxidative stress favors the upregulation and trafficking of AQP4 to the cell surface by a mechanism involving the H_2_O_2_-induced tyrosine phosphorylation of caveolin-1. An altered pattern of AQP expression triggered by oxidative stress was also observed in three cellular models of breast cancer, suggesting the involvement of these membrane proteins in cancer aggressiveness [61].

Since peroxiporin-mediated H_2_O_2_ signaling has been demonstrated to be involved in cancer progression, proliferation, and metastasis [51], in this study, we evaluated the effect of AQP6 silencing in the proliferation of MPM cells. AQP6 reduction by esiRNA treatment was found to suppress MPM cancer proliferation by about 50%. Similar results have been reported on the reduced proliferation of cancer cell lines by silencing the peroxiporins AQP3, AQP5, and AQP8 [62,63,64]. Furthermore, the conditions of oxidative stress, similar to those that occur as a result of the treatment with common chemotherapy treatments, were able to further reduce proliferation, especially in mesothelioma cells.

## 5. Conclusions and Perspectives

In summary, we provide evidence on the role of AQP6 in cancer cell survival and proliferation. The functional role of AQP6 in driving the H_2_O_2_ efflux from MPM cancer cells could explain the resistance of this tumor to conventional chemotherapy and radiotherapy. Thus, AQP6 represents a promising target for novel treatments, making MPM cells more sensitive to oxidative stress created by conventional chemotherapy and radiotherapy. Our study adds a new piece to the complex puzzle of peroxiporin function in MPM, but a great deal of experimental work will be needed to fully understand the mechanisms. AQP9 expression observed in MPM cells membranes should be carefully considered as a peroxiporin that could work together with AQP6. Although the RNA and protein expression of AQP11 does not change in MPM cells, its presence as a peroxiporin of the endoplasmic reticulum and its function in controlling the redox state of the cells should be investigated.

## Figures and Tables

**Table 1:** Primer sequences used for real-time reverse transcription/polymerase chain reaction.

Gene	Primer Sequences		Size (bp)	Accession Number
AQP2 ^a^	Forward	5′-CACCTCCTTGGGATCCATTACACC-3′	95	NM_000486
	Reverse	5′-ACCCAGTGGTCATCAAATTTGCC-3′		
AQP3 ^b^	Forward	5′-CCTGGTGATGTTTGGCTGTGGCTC-3′	147	NM_004925; variants 1, 2
	Reverse	5′-TTCAGGTGGGCCCCAGAGACC-3′		
AQP4	Forward	5′-GGAGTCACCATGGTTCATGGAA-3′	123	NM_001650; variants 1–3
	Reverse	5′-AGTGACATCAGTCCGTTTGGAA-3′		
AQP5	Forward	5′-GGTGGTGGAGCTGATTCTGA-3′	142	NM_001651
	Reverse	5′-GAAGTAGATTCCGACAAGGTGG-3′		
AQP6	Forward	5′-CACCTCATTGGGATCCACTTC-3′	103	NM_ 001652; variants 1,2
	Reverse	5′-CCCAGAAGACCCAGTGGACT-3′		
AQP7	Forward	5′-GGACAGCTGATGGTGACCGG-3′	104	NM_001170; variants 1–4
	Reverse	5′-AGCCACGCCTCATTCAGGAA-3′		
AQP8	Forward	5′-TGGAGAGATAGCCATGTGTGAG-3′	106	NM_001169
	Reverse	5′-TGGCTGCACAAACCGTTCGT-3′		
AQP9	Forward	5′-CCCAGCTGTGTCTTTAGCAA-3′	133	NM_020980; variants 1–3
	Reverse	5′-AAGTCCATCATAGTAAATGCCAAA-3′		
AQP10	Forward	5′-CCTATGTTCTCTACCATGATGCCC-3′	137	NM_080429
	Reverse	5′-CTGATCCAGGAAGCCATTGTTC-3′		
AQP11	Forward	5′-TTTCTCTTCCACAGCGCTCT-3′	115	NM_173039; variant 1
	Reverse	5′-CTCCTGTTAGACTTCCTCCTGC-3′		
β-actin	Hs_ACTB_1_SG, QuantiTect Primer Assay QT00095431, Qiagen		146	NM_001101
B2M	Hs_B2M_1_SG QuantiTect Primer Assay QT00088935, Qiagen		98	NM_004048

Melting temperature, 60 °C; ^a^, 62 °C; ^b^, 66 °C.

## Data Availability

Not applicable.

## Supplementary Materials

The following supporting information can be downloaded at: https://www.mdpi.com/article/10.3390/cells11121892/s1, Figure S1: Immunocytochemical localization of AQP8 protein in MeT5A, REN and MSTO-211H cell lines; Figure S2: Immunocytochemical negative control of MeT5A, REN and MSTO-211H cell lines; Figure S3: Immunofluorescence negative control; Figure S4: Representative curve of stopped-flow osmotic permeability measurement obtained from MeT-5A cell line; Figure S5: AQP6 silencing in mesothelial (A) (MeT-5A), (B) epithelioid (REN), and (C) biphasic (MSTO-211H) MPM cells; Figure S6: qRT-PCR reaction analysis of AQP1, AQP2, AQP3, AQP4, AQP5, AQP7, AQP8, AQP9, AQP10, and AQP11 expression in MSTO-211H cell line silenced for AQP6 (siRNA) and mock-transfected (Ctr); Figure S7: Effect of AQP3 (A) and AQP5 (B) silencing on water permeability of MeT-5A, REN, and MSTO-211H cells; Figure S8: Effect of acid pH on water permeability of MeT-5A, REN, and MSTO-211H.
