# Supplementary material for: Aquaporin-6 May Increase the Resistance to Oxidative Stress of Malignant Pleural Mesothelioma Cells
Source: Cells. 2022 Jun 10;11(12):1892. doi: 10.3390/cells11121892 (PMC9221246; doi:10.3390/cells11121892)
Supplement: Supplementary file 1 [file cells-11-01892-s001.zip › cells-1725843-supplementary.pdf]

Supplementary material

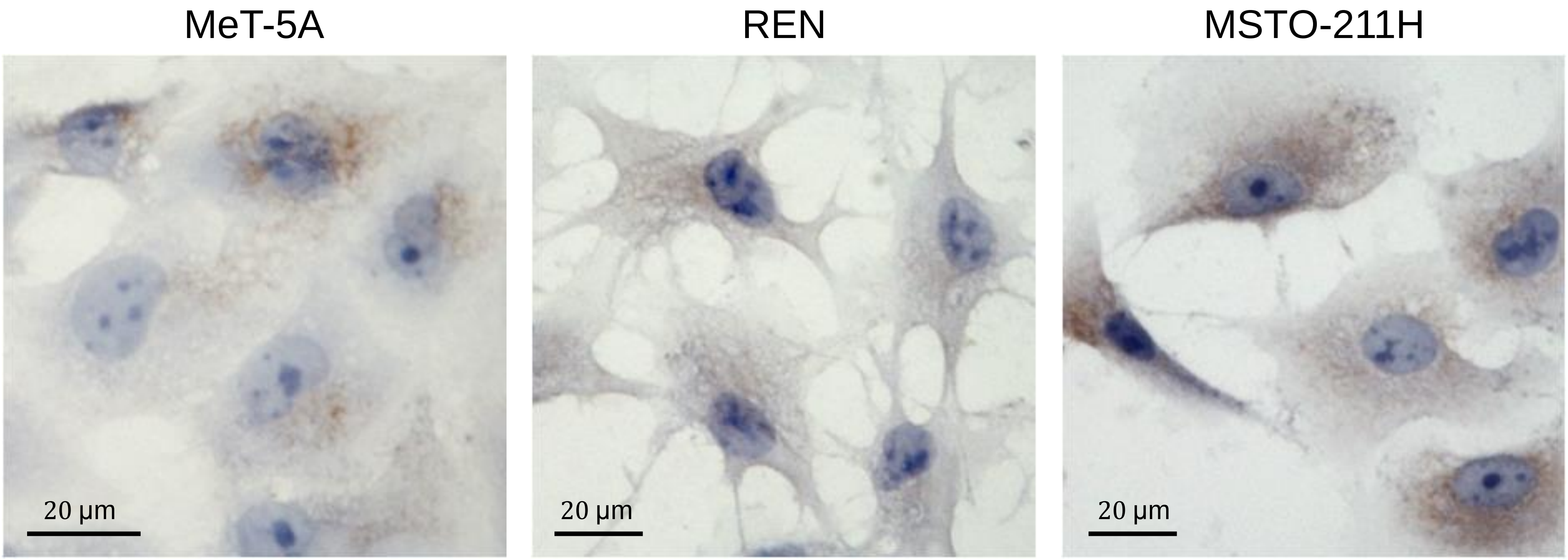

Supplementary Figure S1 - Immunocytochemical localization of AQP8 protein in MeT5A, REN and MSTO-211H cell lines. The protein expression was nil or negligible. Scale bar, 20 µm.

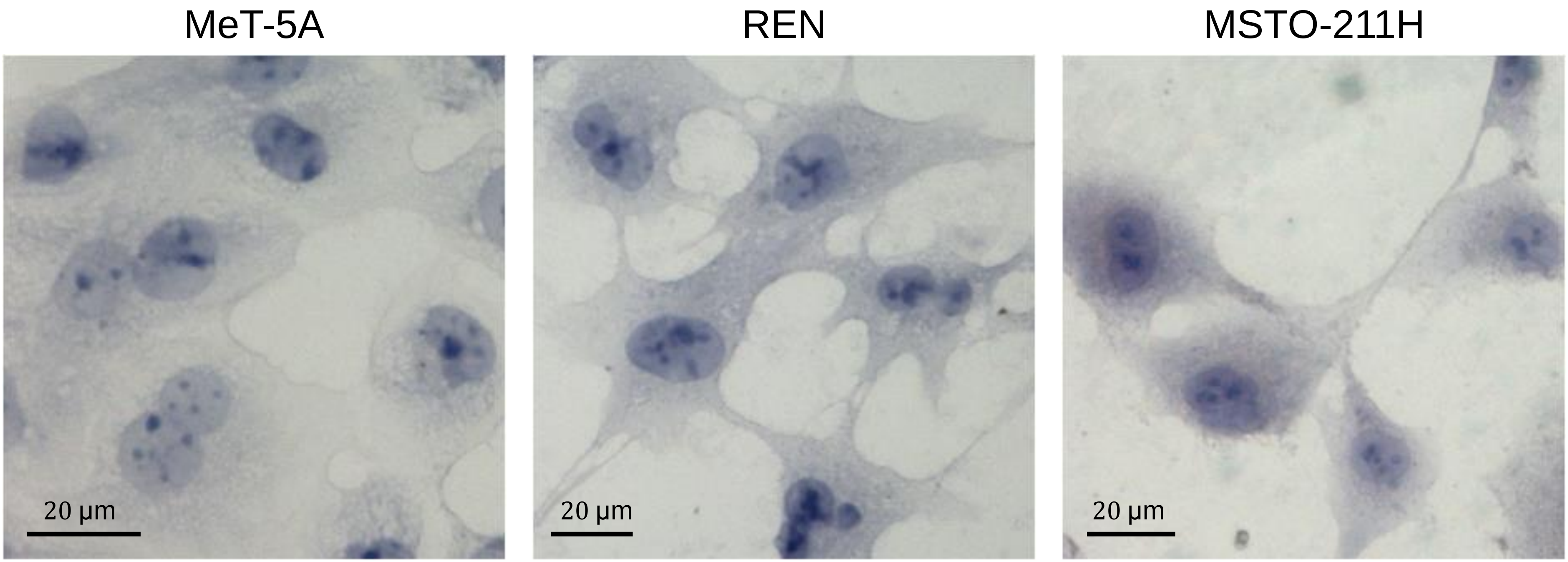

Supplementary Figure S2 - Immunocytochemical negative control of MeT5A, REN and MSTO-211H cell lines. The primary antibody was substituted with non-immune serum. Scale bar, 20 μm.

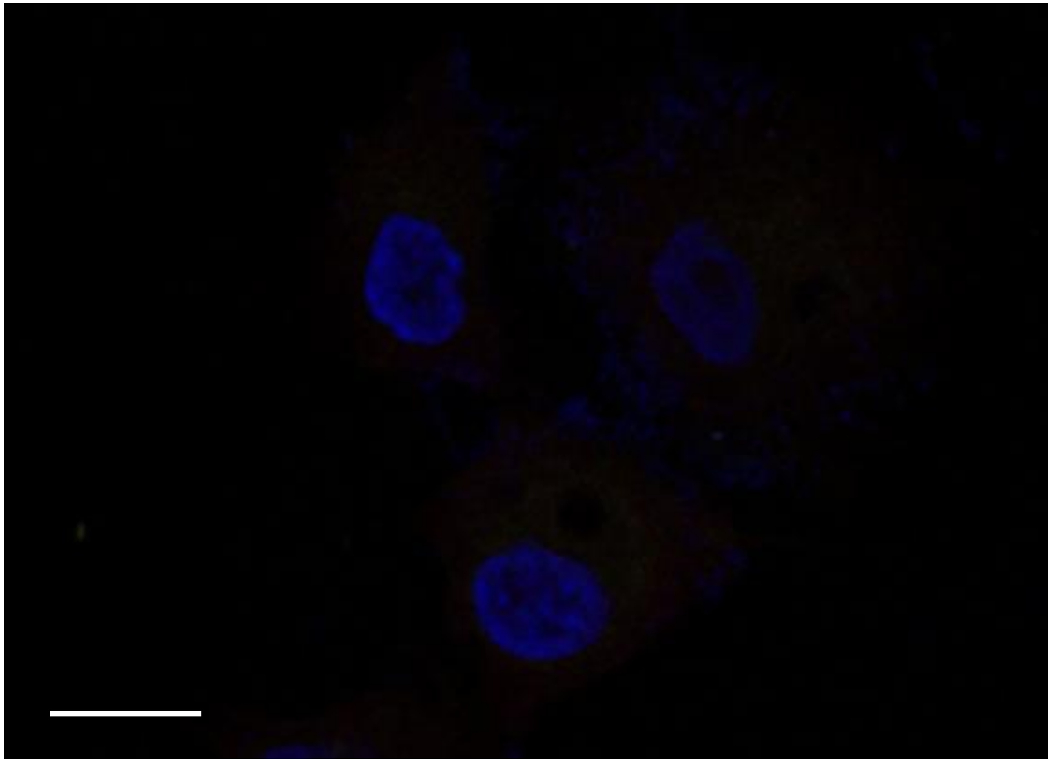

Supplementary Figure S3 - Immunofluorescence negative control in MSTO-211H cell line. No or faint staining was observed when anti-aquaporin-6 and anti-Conacavalin A antibodies were substituted with preimmune serum. Nuclei were counterstained by DAPI (blue). Scale bar, 20 µm.

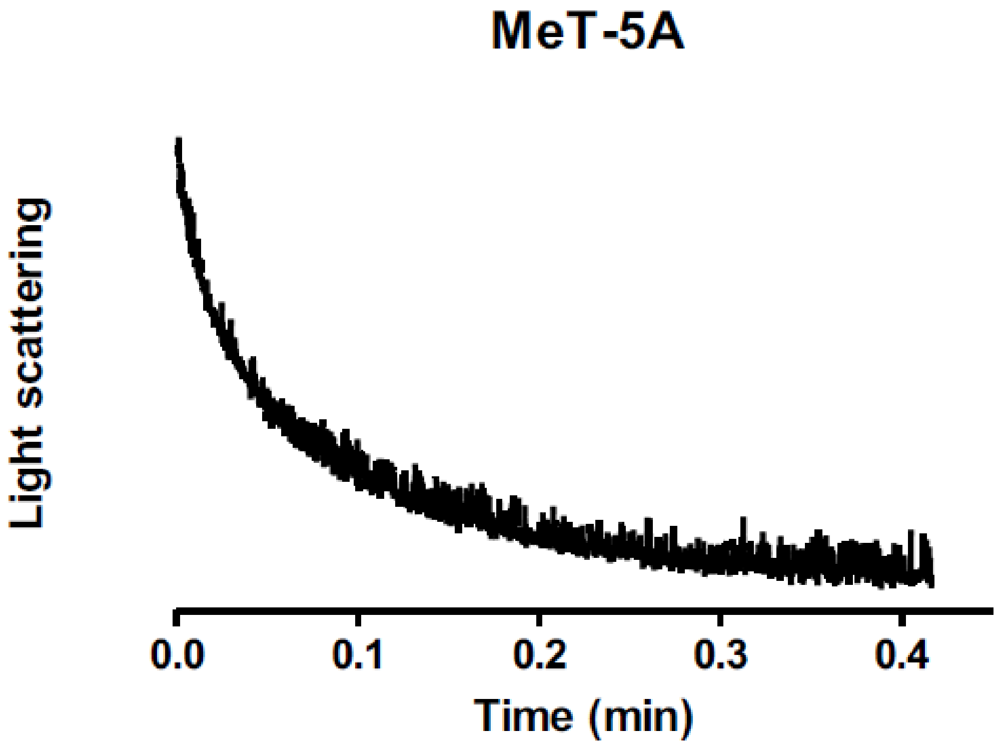

Supplementary Figure S4 - Representative curve of stopped flow osmotic permeability measurement obtained from MeT-5A cell line. Cells were exposed to 150 mOsm hypotonic gradient.

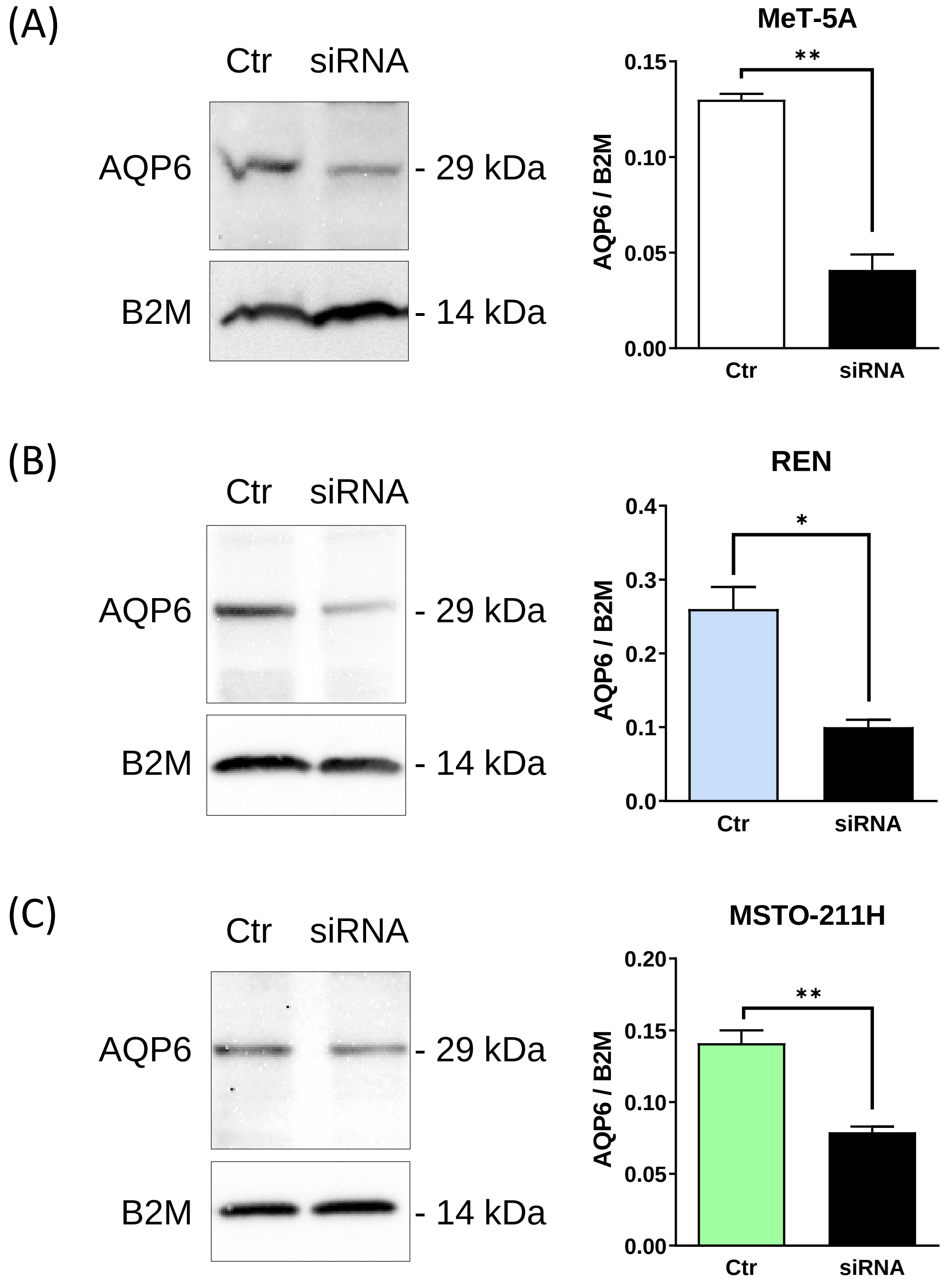

Supplementary Figure S5 - AQP6 silencing in mesothelial (A) (MeT-5A), (B) epithelioid (REN) and (C) biphasic (MSTO-211H) MPM cells. Blots representative of three were shown. Lanes were loaded with 30 µg of proteins, then probed with anti-AQP6 rabbit polyclonal antibody and managed as described in the Materials and Methods. The same blots were stripped and re-probed with anti-β2 microglobulin (B2M) antibody, as housekeeping. Densitometry was performed by acquiring the blot with the iBright CL1000 imaging system. Semiquantitation was performed by iBA (iBright Analysis Software) and the results were normalized to the corresponding B2M.
Densitometry demonstrated a significant reduced protein expression in silenced (siRNA) cells compared to controls (scrambled; Ctr) (*, $p < 0.05$; **, $p < 0.01$; Student's *t* test).

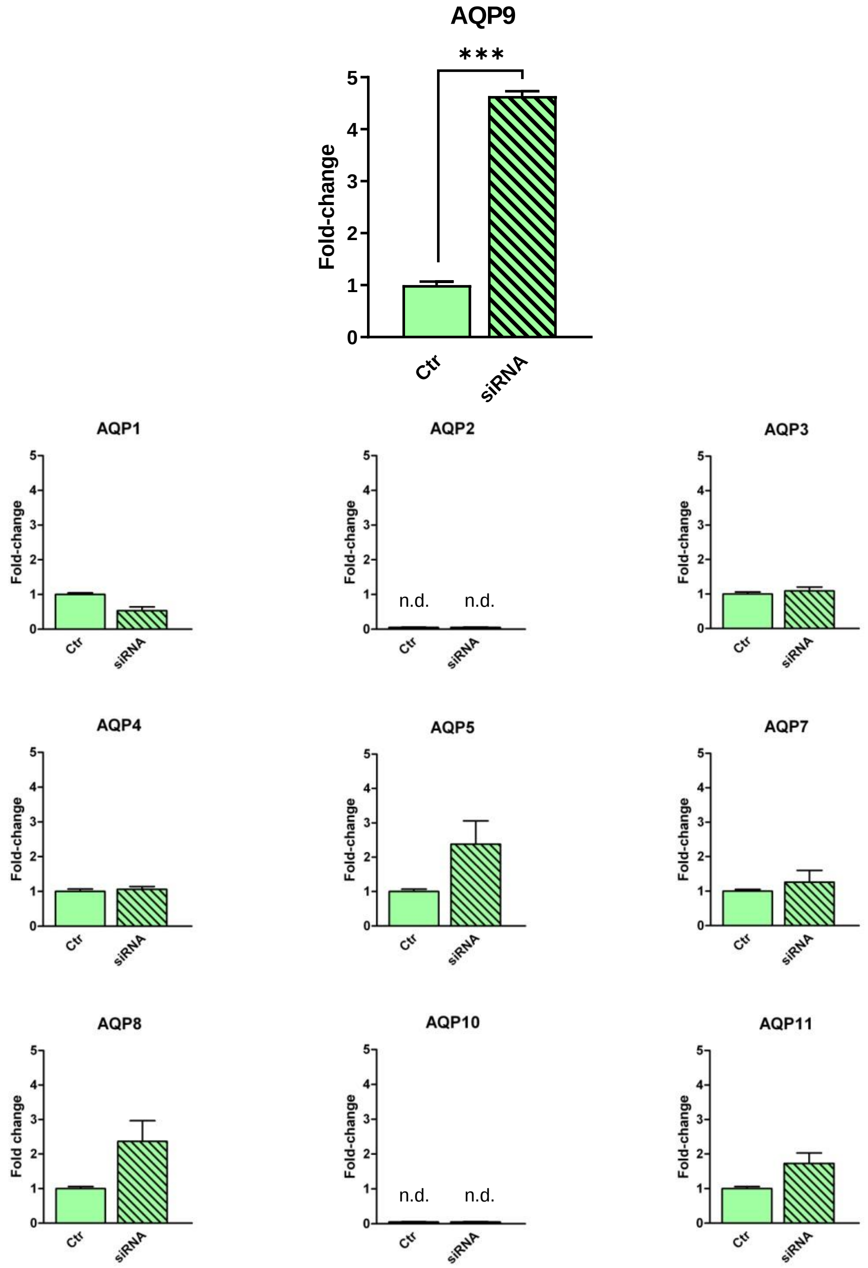

Supplementary Figure S6 - qRT-PCR reaction analysis of AQP1, AQP2, AQP3, AQP4, AQP5, AQP7, AQP8, AQP9, AQP10 and AQP11 expression in MSTO-211H cell line silenced for AQP6 (siRNA) and mock transfected (Ctr). N.d., not detected. Bars represent mean ± S.E.M. of Fold-change values (n = 4). ***: $p < 0.001$ (Student's *t* test).

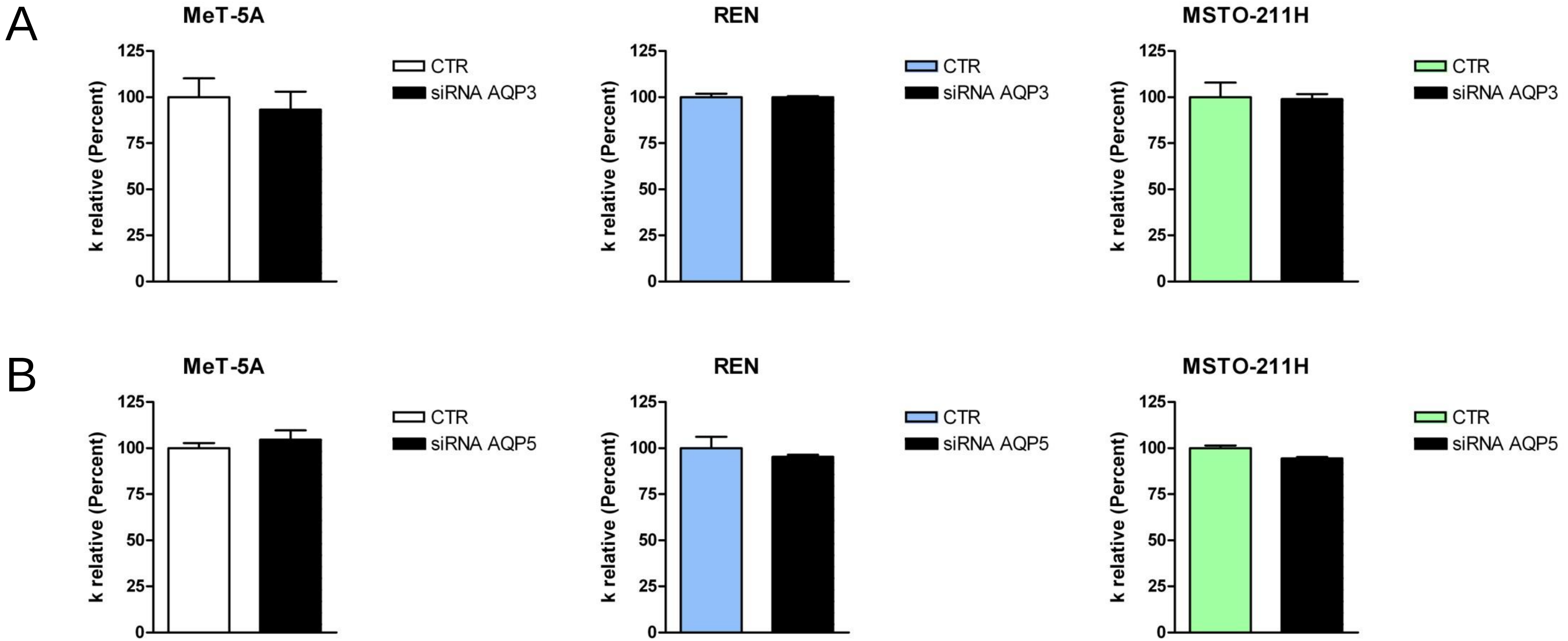

Supplementary Figure S7 - Effect of AQP3 (A), AQP5 (B) silencing on water permeability of MeT-5A, REN, and MSTO-211H cells. Control (CTR) and silenced cells (siRNA AQP) were exposed to a 150 mOsm osmotic gradient. Bars represent the osmotic water permeability of cells expressed as a percent of k relative. Values are means ±S.E.M. of 4–15 single shots for each of 4 different experiments.

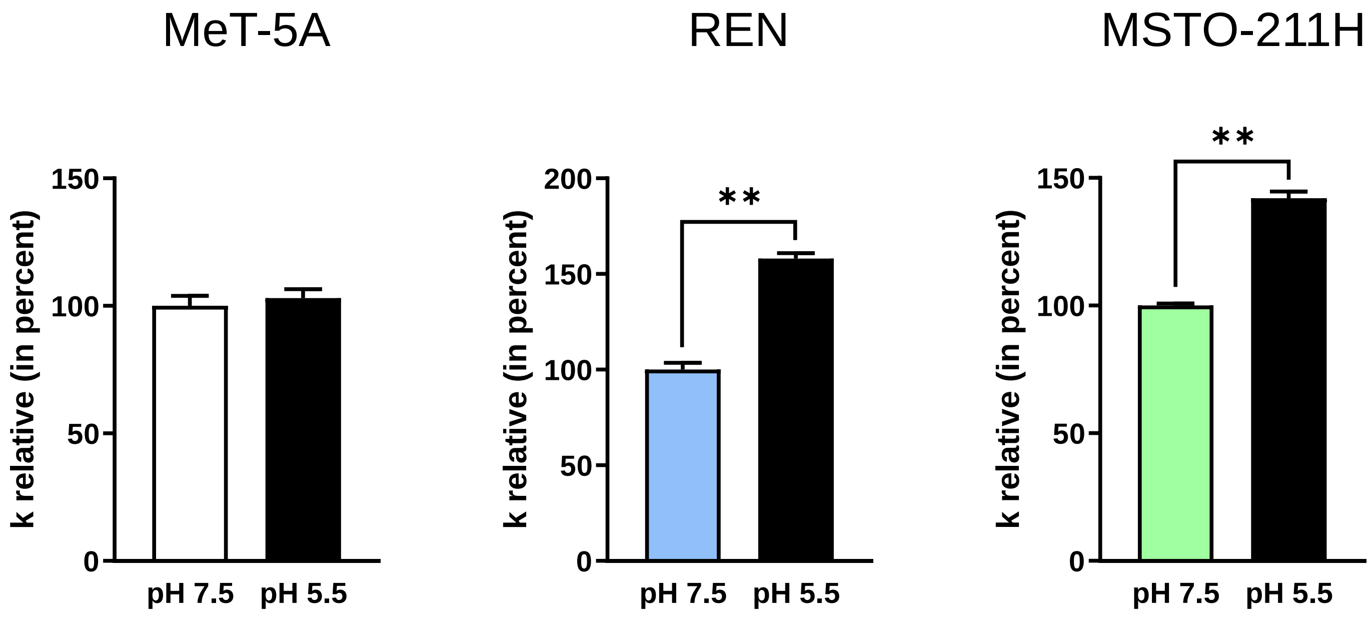

Supplementary Figure S8 - Effect of acid pH on water permeability of MeT-5A, REN, MSTO-211H. Cells were exposed to 150 mOsm osmotic gradient in two different condition: Cell resuspended in PBS pH 7.5 (pH 7.5) and in PBS pH 5.5 (pH 5.5). Bars represent the osmotic water permeability of cells expressed as percent of k relative. Values are means ±S.E.M. of 4 -15 single shots for each of 3 different experiments (*, $p < 0.01$, Student's *t*-test).
